# An accessory renal aneurysm in a patient with absent renal artery: a case report

**DOI:** 10.3389/fcvm.2024.1477604

**Published:** 2024-10-07

**Authors:** Jianghong Wan, Chu Wen Chen, Guoxin Chen, Bin Huang

**Affiliations:** ^1^ Department of Outpatient, West China Hospital, Sichuan University, Chengdu, Sichuan, China; ^2^ Division of Vascular Surgery, Department of General Surgery, West China Hospital, Sichuan University, Chengdu, Sichuan, China

**Keywords:** renal artery aneurysm, accessory renal artery, hypertension, embolization, fibromuscular dysplasia

## Abstract

**Introduction:**

This study reports a patient who developed a secondary renal artery aneurysm (RAA) after occlusion of the main renal artery.

**Methods:**

A 25-year-old woman was hospitalized due to an enlarged renal artery aneurysm (RAA). Computed tomography angiography revealed a 2.2 mm left renal aneurysm and the absence of the left renal artery trunk, with collateral blood supply from the branch arteries of the aorta. The left kidney function remained normal, allowing successful aneurysm embolization. Three years after embolization, the patient's hypertension improved and became more manageable.

**Conclusion:**

Compensation through other abdominal aorta branches after renal artery trunk occlusion is rare, and these branches may also lead to the development of aneurysms. Regular monitoring of these patients is essential.

## Introduction

The common causes of renovascular hypertension are renal arteritis, atherosclerosis, and fibromuscular dysplasia (1). Additionally, two-thirds of patients with renal aneurysms also have hypertension, and most patients experience hypertension improvement after renal vascular reconstruction (2). Fibromuscular dysplasia (FMD) is a noninflammatory and nonatherosclerotic arterial disease that mostly affects the renal arteries (3, 4) and occurs predominantly in women aged 20–60 years.

The mechanism of hypertension in both renal aneurysms and FMD is unclear. It is thought that the sympathetic nervous system, with fibers from the aorta located in the adventitia of the renal arteries, is a critical contributor to the regulation of both effects in acute and chronic states and has been proven to be overactivated in all forms of hypertension (5). According to the new RADIANCE Clinical Trial Program (6), ultrasound renal denervation decreases blood pressure in patients with mild to moderate hypertension and hypertension who are resistant to treatment.

Although color ultrasound can now accurately reveal the lesion, the characteristics and anatomic properties of the lesion can be better demonstrated by computed tomography angiography (CTA) or angiography. No cases of renal artery occlusion caused by FMD have been reported. It is very rare to develop collateral circulation to satisfy the renal blood supply and maintain normal renal function after main renal artery occlusion. Moreover, how can one manage an aneurysm located at the proximal end of one of these important branch arteries? This article describes the treatment and results of a patient with a secondary aneurysm in this special condition.

## Case presentation

**Figure 1:**
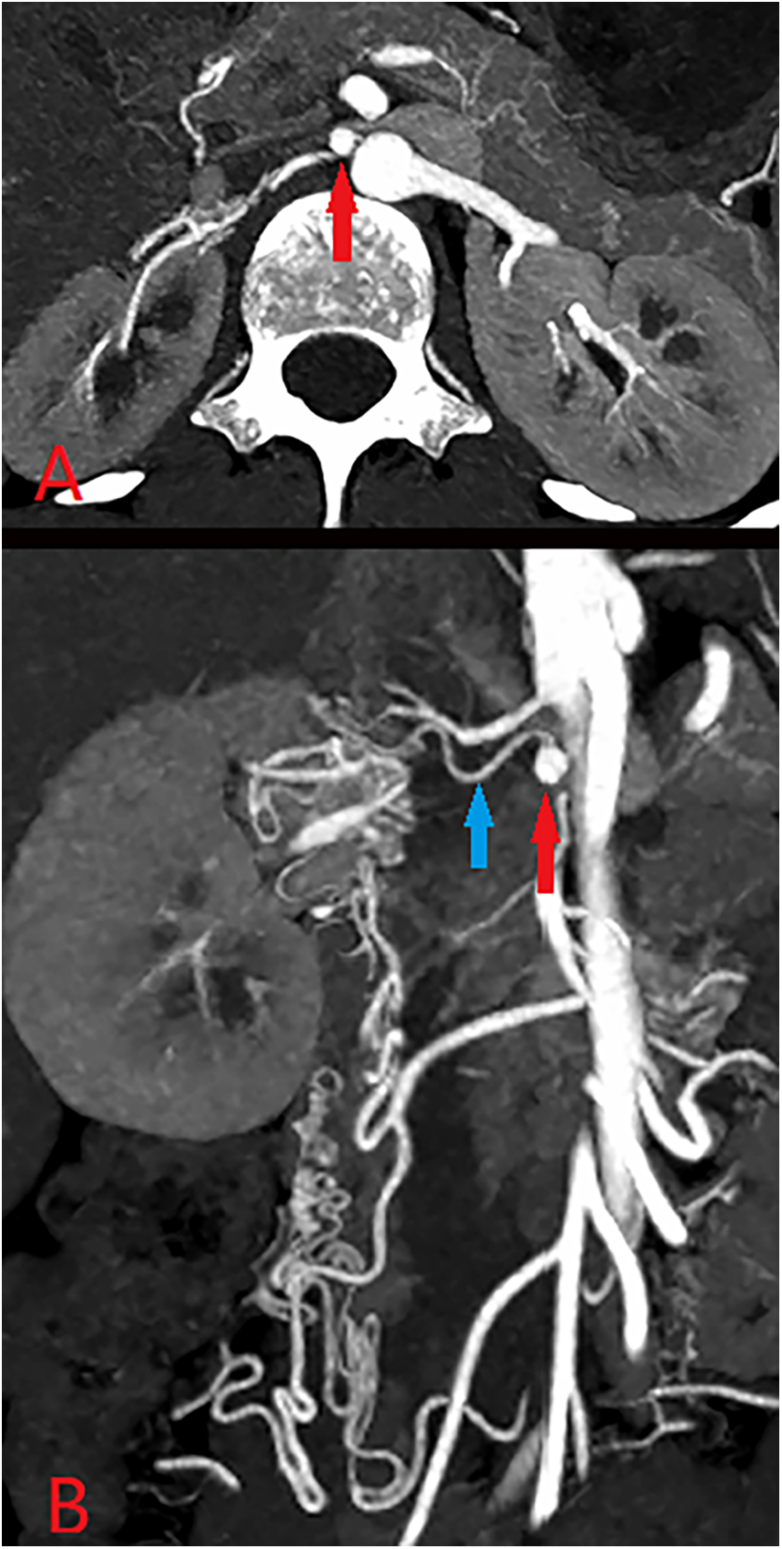
CTA **(A,B)** revealed that the aneurysm (approximately 0.5*0.5 cm, red arrow) was located proximal to the renal artery (blue), and several branch arteries formed the collateral circulation for the right kidney.

A 25-year-old female was admitted with the chief complaint that a renal artery aneurysm (RAA) had been found 7 years prior and had been enlarging for 3 months. Approximately 7 years ago, the patient presented to our hospital because of a systolic pressure of 240 mmHg. CTA revealed several renal arteries supplying the right kidney, and the aneurysm was located at the proximal end of the second renal artery (Figure 1). However, the right main renal artery was not clearly visualized on the CT, raising suspicion of occlusion and degeneration. Furthermore, the right kidney is smaller in volume than the left kidney. Immunological inspection revealed no abnormalities. The glomerular filtration rate (GFR) was 136.60 ml/min/1.73 m^2^. Aldosterone (decubitus) was 59.25 ng/dl, angiotensin (decubitus) was 88.07 ng/dl, norepinephrine was 365 ng/L, and epinephrine was 31 ng/L. Considering that the aneurysm was small (0.5 × 0.5 cm, Figure 1) and that sacrificing the collateral artery during embolization could lead to decreased renal function, close follow-up observations and proactive management of hypertension were recommended.

**Figure 2:**
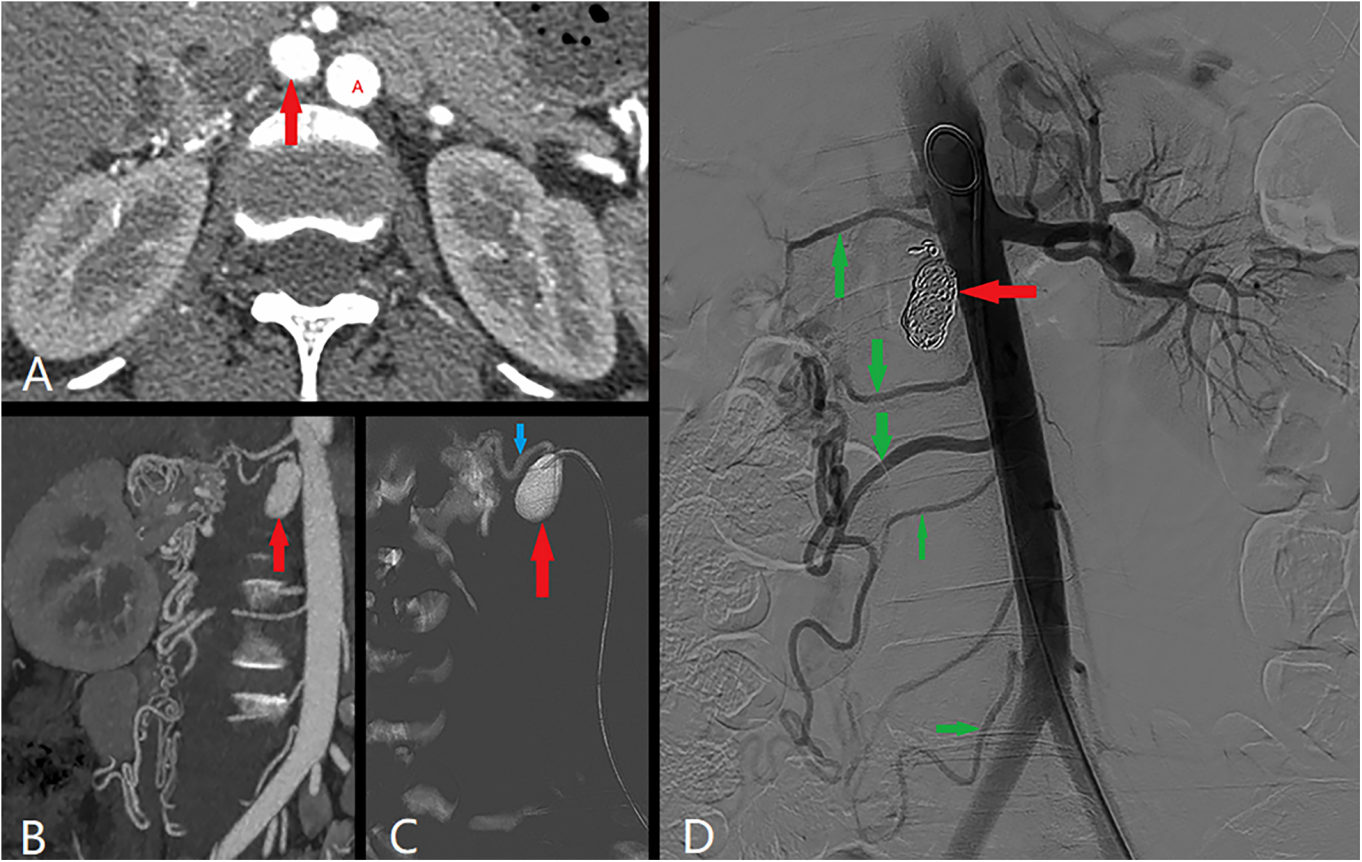
Angiography revealed that the aneurysm was enlarged to 2.20*1.10 cm (red arrow). Red A was abdominal aorta. Angiography revealed that the collateral circulation was composed of the renal artery (blue arrow) and branch arteries (green arrow).

The reason for this hospitalization was that the patient experienced an increase in RAA three months prior. Computed tomography angiography (CTA) revealed that the volume of the identified aneurysm (2.20*1.10 cm, Figures 2A–C) and the kidney (Supplementary Figures 1B,D) increased noticeably compared with that of the CTA in 2013 (Supplementary Figures 1A,C). The collateral arteries of the right kidney appeared to be increasing in size.

Blood pressure (BP) was controlled by the oral administration of labelolol hydrochloride (100 mg, twice a day) and felodipine (5 mg, once a day) and fluctuated between 130/70 mmHg and 160/100 mmHg. Laboratory examination revealed that the serum creatinine level was 61 µmol/L, and the GFR was 129.72 ml/min/1.73 m^2^. SPECT renal imaging revealed that the right renal GFR (ml/min) was 44.51, and the left renal GFR was 38.37. No positive inflammatory indicators or abnormalities in immunologic function were detected. Venous blood samples taken at the level of the renal veins revealed the following values: in the left renal vein, the renin level was approximately 1.32 µIU/ml, the aldosterone level was approximately 7.00 ng/dl, whereas in the right renal vein, the renin level was approximately 1.90 µIU/ml, and the aldosterone level was approximately 6.76 ng/dl. After fully understanding the patient's condition and treatment plan, the patient chose embolization treatment.

Angiography confirmed the presence of the branch aneurysm and the absence of the main renal artery. The kidney was supplied by other branches originating from the abdominal aorta. The aneurysm was subsequently embolized via COOK coils of various sizes (10 mm, 8 mm, and 6 mm, totaling 13 coils). Postsurgery, hydration and antiplatelet therapy were administered as part of routine care. The serum creatinine level was 61 µmol/L, with an estimated glomerular filtration rate (eGFR) of 121.38 ml/min/1.73 m^2^. Blood pressure was successfully maintained at approximately 140/85 mmHg.

The patient was discharged successfully. After three months, the dosage of labetalol was reduced by half, and after six months, the blood pressure stabilized and remained stable. Three years later, the patient was pleased to report that only felodipine (2.5 mg, once a day) was needed to control her blood pressure well.

## Discussion

Fibromuscular dysplasia (FMD) can lead to arterial stenosis, occlusion, aneurysm, dissection and tortuosity and can cause hypertension. FMD lesions are typically located away from the origin of the renal artery, often in the midportion of the vessel or at the first arterial bifurcation (7). Additionally, there was a mean delay from the onset of FMD-mediated hypertension to FMD diagnosis of 4 years for unifocal FMD and 9 years for multifocal FMD (MFMD) (8). The current mostly accepted mechanism for renovascular hypertension is that a reduction in blood flow perfusion to the kidney causes alterations in the renin‒angiotensin‒aldosterone system (9). This has also been demonstrated in our case. However, whether this is related to the renal sympathetic nerve is unknown.

With the establishment and development of collateral circulation, blood flow to the right kidney increases, causing its volume to gradually expand until it is comparable to that of the left kidney, leading to a gradual normalization of the secretion levels of renin and angiotensin. Interestingly, neither unifocal FMD nor MFMD has been reported in current studies to cause renal artery occlusion. Even rarer patients maintain normal renal function after experiencing arterial occlusion. Dobrzinski et al. (10) reported a 47-year-old female who was found to have occlusion of the left renal artery accompanied by decreased left kidney function due to hypertension. After one year of medical treatment, the left kidney returned to normal function through collateral circulation, and the hypertension became easier to control.

In addition, no clear evidence has revealed the relationship between ARA stenosis and HTN (11, 12). However, Calinoiu et al. (13) reported that accessory renal artery stenosis led to a significant increase in renin levels, causing hypertension. Generally, the diameters of the main renal arteries were significantly smaller when the ARA was present. The main renal artery is considered to be a single, dominant artery arising from the abdominal aorta that enters the renal hilum and branches, whereas the ARAs do not branch prior to entry into the renal parenchyma (14). ARAs can affect 25%–50% of patients and originate from the abdominal aorta, the main renal artery, other branches of the aorta, etc. The number of AAs vastly differs between individuals because of the complex nature of renal embryogenesis (15).

This condition could be attributed to congenital dysplasia of the renal arteries. However, this patient was not considered congenital because her symptoms did not begin until she was an adult. A diagnosis of fibromuscular dysplasia (FMD) is reasonable given the changes in renin and angiotensin levels, the presence of main renal artery occlusion, the absence of immunological markers, the absence of renal artery calcification, and the lack of a specific family history. For these patients, routine renal artery screening should be conducted and reviewed annually.

Notably, despite the occlusion of the main right renal artery, the patient's right kidney continued to grow into adulthood until it was similar in size to the left kidney. These positive changes render the carrier artery no longer strategically advantageous. Interestingly, hypertension improved following aneurysm embolization. This may be attributable to changes in pressure within the collateral circulation network following embolization, leading to a redistribution of blood flow and an increased blood supply to the kidney.

## Conclusion

Compensation through other branches of the abdominal aorta after occlusion of the renal artery trunk is very rare. Moreover, these branches may also subsequently lead to aneurysms. Regular monitoring is necessary for these patients. The choice of treatment should still be determined on the basis of the individual patient's condition.

## Data Availability

The original contributions presented in the study are included in the article/Supplementary Material, further inquiries can be directed to the corresponding author.

## Supplementary material

Supplementary Figure S1 Compared with 7 years ago, the volume difference between the two kidneys decreased significantly. (The right kidney was larger, and the left kidney was smaller. **A** and **C** was in 2013. **B** and **D** was in 2020).
