# Supplementary figures and images for: An accessory renal aneurysm in a patient with absent renal artery: a case report
Source: Front Cardiovasc Med. 2024 Oct 7;11:1477604. doi: 10.3389/fcvm.2024.1477604 (PMC11494606; doi:10.3389/fcvm.2024.1477604)

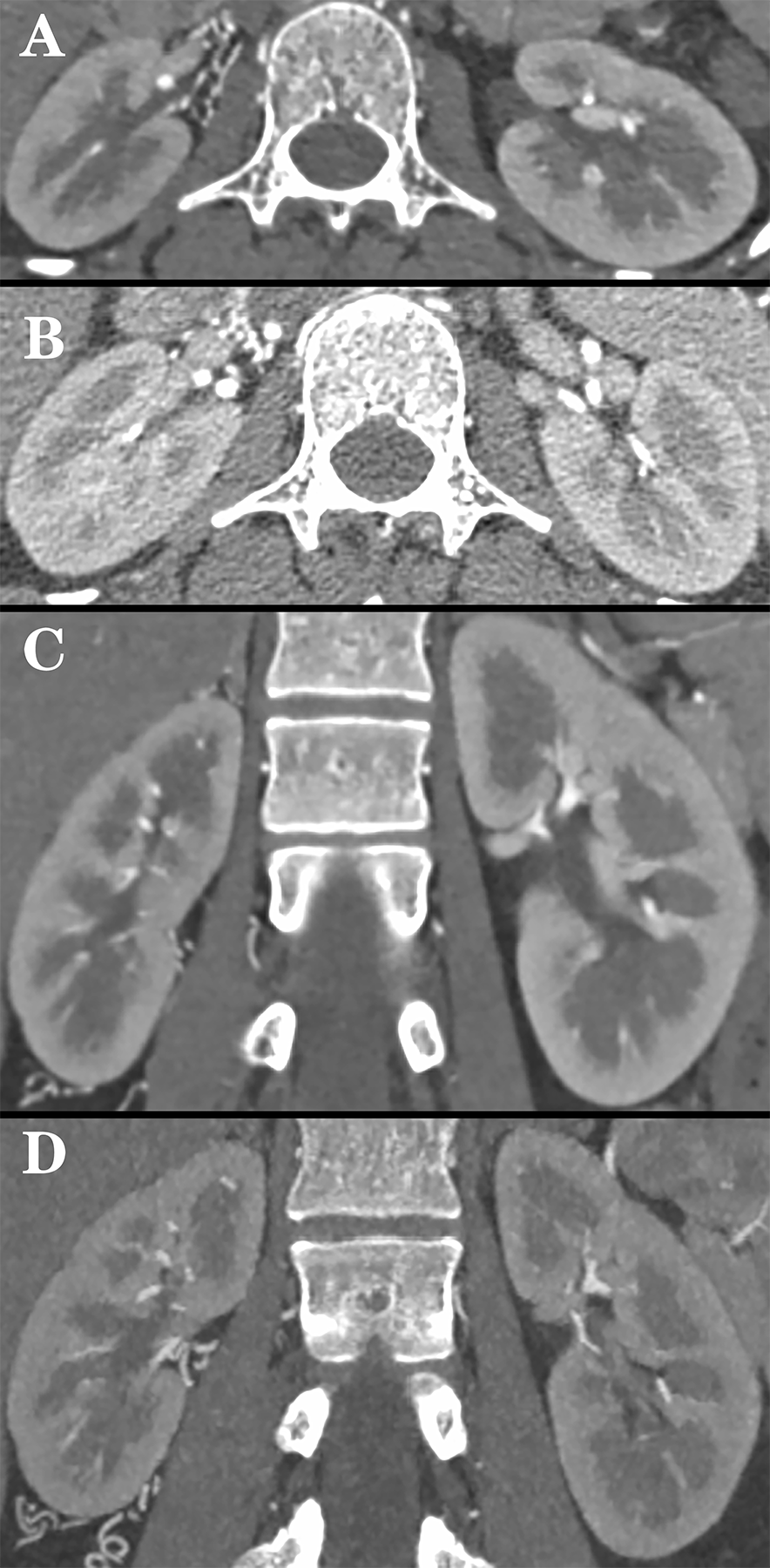

Supplement: Supplementary Figure S1 [file Image1.tif]

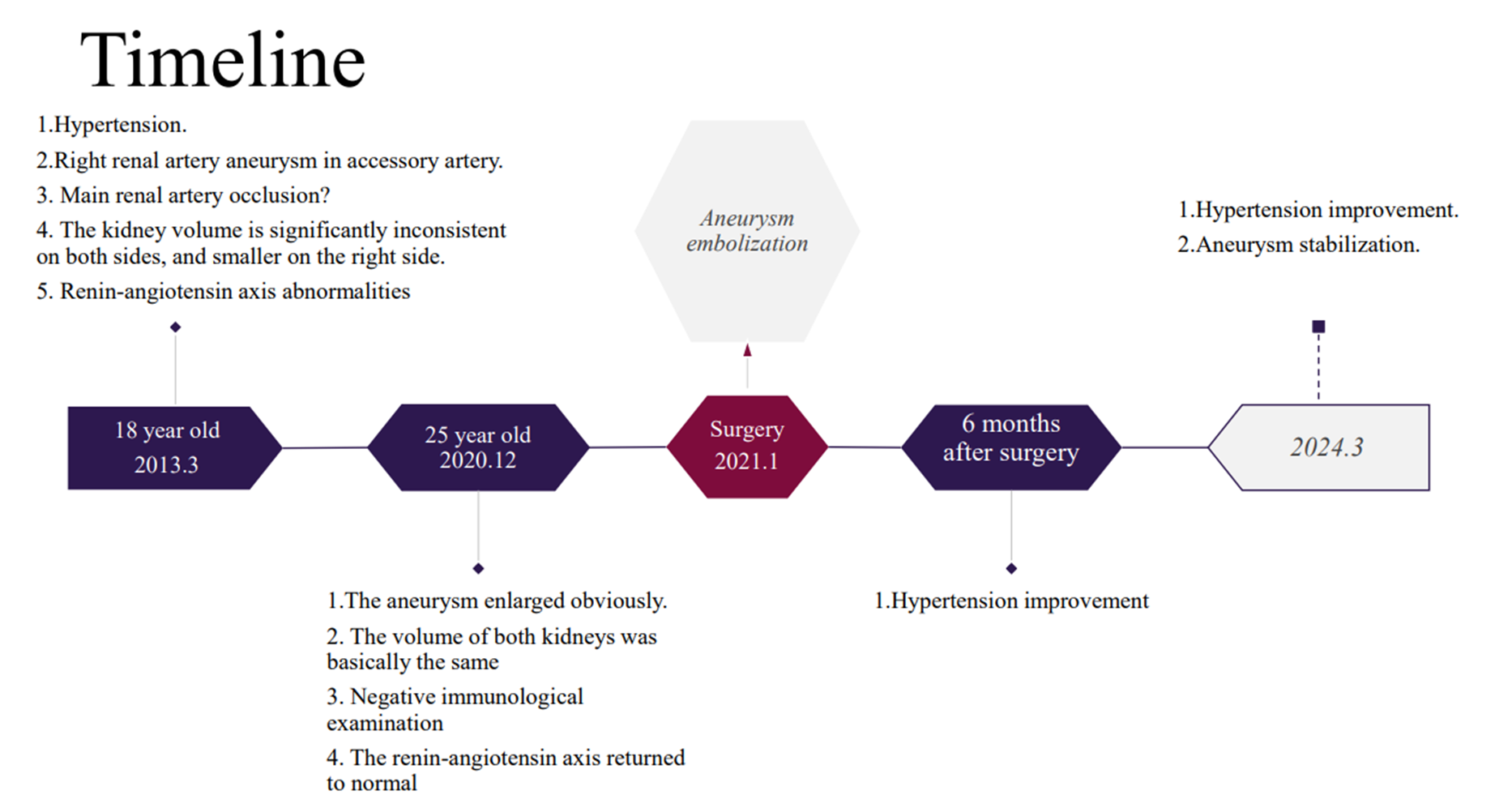

Supplement: Supplementary file 2 [file Image2.tif]
